# Testing the generalizability of ancestry-specific polygenic risk scores to predict prostate cancer in sub-Saharan Africa

**DOI:** 10.1186/s13059-022-02766-z

**Published:** 2022-09-13

**Authors:** Michelle S. Kim, Daphne Naidoo, Ujani Hazra, Melanie H. Quiver, Wenlong C. Chen, Corinne N. Simonti, Paidamoyo Kachambwa, Maxine Harlemon, Ilir Agalliu, Shakuntala Baichoo, Pedro Fernandez, Ann W. Hsing, Mohamed Jalloh, Serigne M. Gueye, Lamine Niang, Halimatou Diop, Medina Ndoye, Nana Yaa Snyper, Ben Adusei, James E. Mensah, Afua O. D. Abrahams, Richard Biritwum, Andrew A. Adjei, Akindele O. Adebiyi, Olayiwola Shittu, Olufemi Ogunbiyi, Sikiru Adebayo, Oseremen I. Aisuodionoe-Shadrach, Maxwell M. Nwegbu, Hafees O. Ajibola, Olabode P. Oluwole, Mustapha A. Jamda, Elvira Singh, Audrey Pentz, Maureen Joffe, Burcu F. Darst, David V. Conti, Christopher A. Haiman, Petrus V. Spies, André van der Merwe, Thomas E. Rohan, Judith Jacobson, Alfred I. Neugut, Jo McBride, Caroline Andrews, Lindsay N. Petersen, Timothy R. Rebbeck, Joseph Lachance

**Affiliations:** 1 School of Biological Sciences, Georgia Institute of Technology, 950 Atlantic Dr, Atlanta, GA 30332 USA; 2 Centre for Proteomic and Genomic Research, Cape Town, South Africa; 3 Sydney Brenner Institute for Molecular Bioscience, Faculty of Health Sciences, University of the Witwatersrand, Johannesburg, South Africa; 4 National Cancer Registry, National Health Laboratory Service, Johannesburg, South Africa; 5 Department of Epidemiology and Population Health, Albert Einstein College of Medicine, Bronx, NY USA; 6 University of Mauritius, Réduit, Mauritius; 7 Faculty of Medicine and Health Sciences, Stellenbosch University, Cape Town, South Africa; 8 Stanford Cancer Institute, Stanford University, Stanford, CA USA; 9 Universite Cheikh Anta Diop de Dakar, Dakar, Senegal; 10 37 Military Hospital, Accra, Ghana; 11 Korle-Bu Teaching Hospital and University of Ghana Medical School, Accra, Ghana; 12 Department of Pathology, University of Ghana Medical School, Accra, Ghana; 13 College of Medicine, University of Ibadan, Ibadan, Nigeria; 14 College of Health Sciences, University of Abuja and University of Abuja Teaching Hospital, Abuja, Nigeria; 15 Non-Communicable Diseases Research Division, Wits Health Consortium (PTY) Ltd, Johannesburg, South Africa; 16 MRC Developmental Pathways to Health Research Unit, Department of Pediatrics, Faculty of Health Sciences, University of Witwatersrand, Johannesburg, South Africa; 17 Keck School of Medicine, University of Southern California, Los Angeles, CA USA; 18 Herbert Irving Comprehensive Cancer Center, Columbia University, New York, NY USA; 19 Dana-Farber Cancer Institute, Boston, MA USA; 20 Harvard T.H. Chan School of Public Health, Boston, MA USA

**Keywords:** Africa, Health disparities, Genomic medicine, Polygenic risk scores, Population genetics, Prostate cancer

## Abstract

**Background:**

Genome-wide association studies do not always replicate well across populations, limiting the generalizability of polygenic risk scores (PRS). Despite higher incidence and mortality rates of prostate cancer in men of African descent, much of what is known about cancer genetics comes from populations of European descent. To understand how well genetic predictions perform in different populations, we evaluated test characteristics of PRS from three previous studies using data from the UK Biobank and a novel dataset of 1298 prostate cancer cases and 1333 controls from Ghana, Nigeria, Senegal, and South Africa.

**Results:**

Allele frequency differences cause predicted risks of prostate cancer to vary across populations. However, natural selection is not the primary driver of these differences. Comparing continental datasets, we find that polygenic predictions of case vs. control status are more effective for European individuals (AUC 0.608–0.707, OR 2.37–5.71) than for African individuals (AUC 0.502–0.585, OR 0.95–2.01). Furthermore, PRS that leverage information from African Americans yield modest AUC and odds ratio improvements for sub-Saharan African individuals. These improvements were larger for West Africans than for South Africans. Finally, we find that existing PRS are largely unable to predict whether African individuals develop aggressive forms of prostate cancer, as specified by higher tumor stages or Gleason scores.

**Conclusions:**

Genetic predictions of prostate cancer perform poorly if the study sample does not match the ancestry of the original GWAS. PRS built from European GWAS may be inadequate for application in non-European populations and perpetuate existing health disparities.

**Supplementary Information:**

The online version contains supplementary material available at 10.1186/s13059-022-02766-z.

## Background

Prostate cancer (CaP) has a complex etiology, with substantial contributions from inherited genetic factors [1–3]. Among men, CaP is the most commonly diagnosed cancer worldwide, but incidence and mortality rates vary across global populations. East Asians have the lowest observed rates of CaP, and Africans and men living in the Caribbean have the highest observed rates [4, 5]. African American men are 1.8 times more likely to be diagnosed with CaP and 2.4 times more likely to die from the disease than European Americans [6, 7]. Some of these differences in risk may be due to genetic causes, including continental differences in allele frequencies at CaP-associated loci [8]. CaP has a heritability of 58% [9, 10], and men who have a first-degree relative with CaP have a higher risk of CaP than men without a family history [9, 11].

Genome-wide association studies (GWAS) have identified hundreds of loci associated with increased risk of CaP [12–20], but most of these loci were discovered in individuals of European descent. Although genetic associations with CaP have been identified in men of African descent [21–24], this relative underrepresentation in GWAS suggests that many CaP-associated loci are as yet undiscovered [25]. Many genotyping arrays use markers that were largely ascertained in non-African populations, thus yielding a biased set of disease associations [26–28]. Moreover, effect sizes at cancer-associated loci can differ by ethnicity and ancestry [29, 30]. Collectively, these issues limit the generalizability of genetic predictions of cancer risk to non-European populations [31–35].

GWAS results can be leveraged to generate polygenic risk scores (PRS), which quantify an individual’s genetic propensity to develop disease [36, 37]. PRS have been effectively used to classify whether individuals of European descent are more likely to develop complex diseases like breast or prostate cancer [38–41]. Future clinical applications of PRS include assisting in diagnosis and informing treatment options [42, 43]. Recently, a trio of well-powered GWAS have yielded risk scores for CaP. Schumacher et al. leveraged data from over 140,000 cases and controls of European ancestry to discover 63 new CaP-associated loci [38]. This led to the generation of a 147-marker PRS [38]. Conti et al. performed a multi-ancestry meta-analysis of over 234,000 cases and controls, finding 83 novel CaP-associated variants and generating a 269 marker PRS [44]. Importantly, the PRS generated by Conti et al. contains ancestry-specific weights [44]. Age of diagnosis information can also be leveraged to generate polygenic hazard scores (PHS), which predict whether individuals are more likely to have early-onset CaP [45]. Karunamuni et al. combined 46 SNPs ascertained in men of European descent with three SNPs that were ascertained in men of African descent to generate the PHS46+African hazard score [46]. These three PRS are denoted here as the Schumacher, Conti, and PHS46+African PRS, respectively. Note that the multi-ancestry Conti PRS builds upon the Schumacher PRS.

Here, we assess the generalizability of CaP PRS using European data from the UK Biobank (UKBB) and a novel African dataset from the *M*en of *A*frican *D*escent and *Ca*rcinoma of the *P*rostate (MADCaP) Network [47]. We investigate the following questions: (1) How much do allele frequencies of CaP-associated loci vary across continental populations? (2) Are these allele frequency differences driven by natural selection? (3) Are existing PRS generalizable to sub-Saharan African (SSA) populations? (4) How much does incorporating ancestry-matched information improve genetic prediction of CaP?

## Results

### Population genetics of MADCaP Network samples

**Fig. 1:**
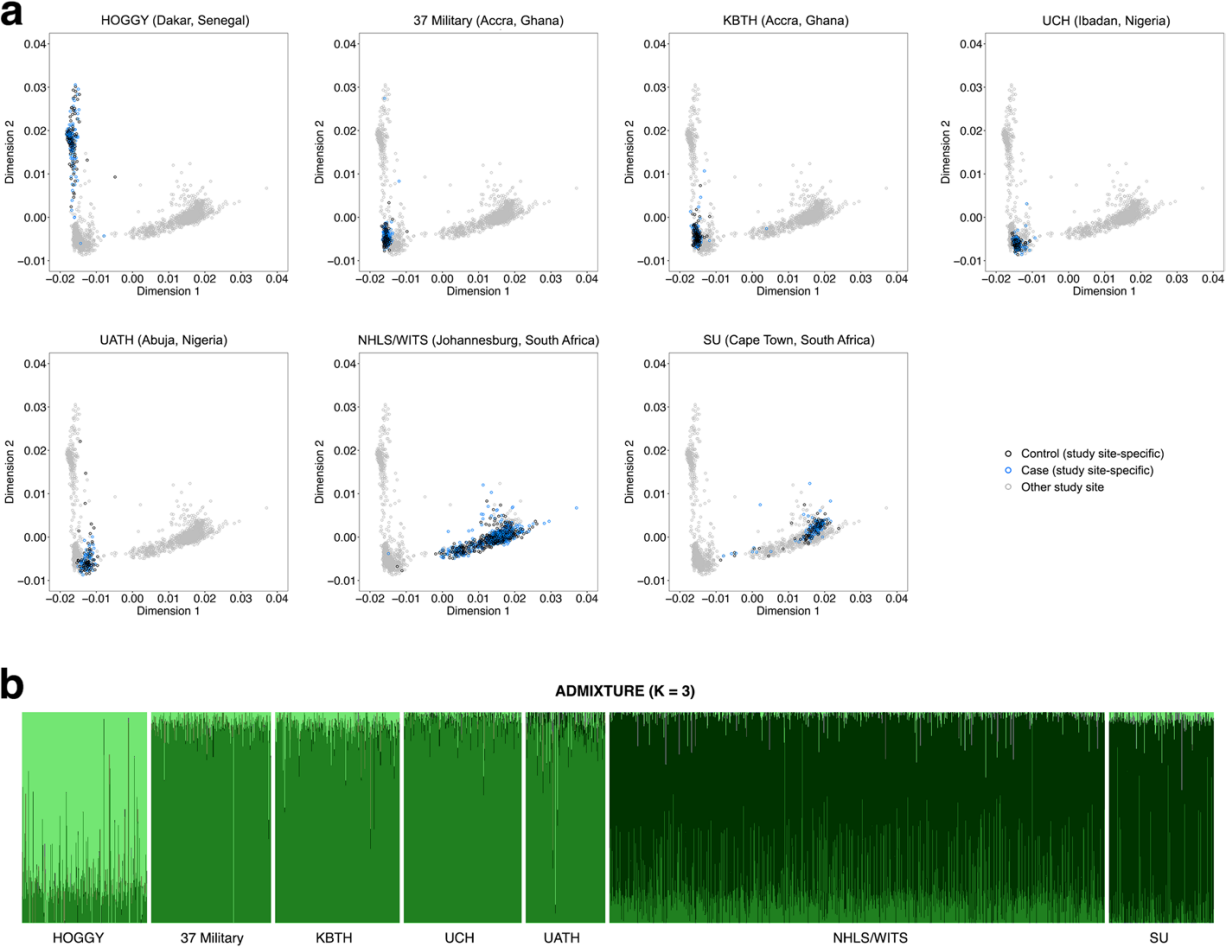
Population structure of MADCaP Network samples reveals shared genetic ancestries among urban and suburban African study sites. **A** Two-dimensional MDS plots of 2631 MADCaP individuals. Subpanels focus on specific study sites, with controls colored black, CaP cases colored blue, and samples from other study sites colored grey. **B** ADMIXTURE plot of 2631 MADCaP individuals. Abbreviations of MADCaP Network study sites are listed in the “Methods” section

African cases and controls were sampled from MADCaP study sites in Senegal, Ghana, Nigeria, and South Africa. Summary statistics of MADCaP samples are described in Table 1. African individuals were recruited from urban and suburban locales [47]. The primary languages spoken by MADCaP participants differ for Senegal (Wolof, Pulaar, and French), Ghana (Akan, Ga-Dangme, Ewe, and English), Nigeria (Yoruba, Igbo, Hausa, and English), and South Africa (isiXhosa, isiZulu, Sesotho, Setswana, English, and Afrikaans). For each MADCaP study site, Fig. 1a shows that cases (blue) and controls (black) cluster together, indicating that cases and controls are ancestry-matched. West African individuals are found on the left of each multidimensional scaling (MDS) plot, and South African individuals are found on the bottom right of each MDS plot (Fig. 1a). This observed population structure is broadly consistent with a pilot study from the MADCaP Network [48]. An ADMIXTURE plot reveals further population structure among MADCaP samples: Senegalese individuals have a different mix of ancestries than Ghanaian, Nigerian, and South African individuals (compare different shades of green for each study site in Fig. 1b).

**Table 1:** Characteristics of SSA cases and controls from the MADCaP Network

MADCaP participant characteristics	Cases (*n*=1298)	Controls (*n*=1333)
African study site		
Hôpital Général de Grand Yoff	136	145
37 Military Hospital	136	142
Korle-Bu Teaching Hospital	144	136
University College Hospital	135	130
University of Abuja Teaching Hospital	91	88
WITS Health Consortium	537	576
Stellenbosch University	119	116
Age in years		
< 70	24.5%	24.3%
70–79	30.2%	39.2%
≥ 80	45.3%	36.5%
Tumor stage		
T1	37.4%	NA
T2	44.6%	NA
T3	10.2%	NA
T4	7.8%	NA
Gleason score		
≤ 6	17.1%	NA
7	43.6%	NA
≥ 8	39.2%	NA

### Evolutionary genetics of CaP-associated loci

**Fig. 2:**
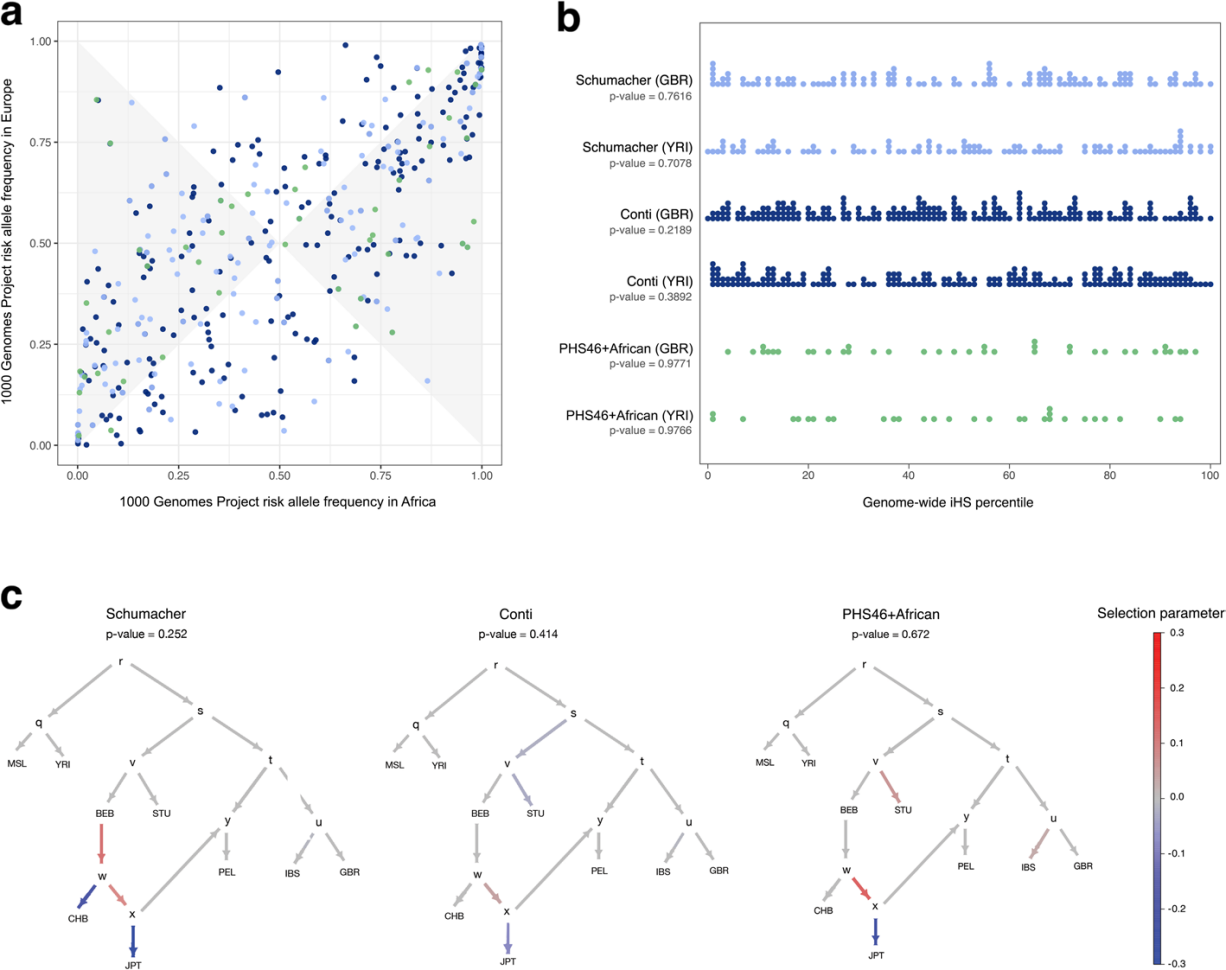
Evolutionary genetics of CaP-associated variants. **A** Joint site frequency spectrum of risk allele frequencies in Europe and Africa (1KGP data). Minor allele frequencies are larger for Europe than Africa in the shaded region. Schumacher PRS variants are denoted by light blue points, Conti PRS variants are denoted by dark blue points, and PHS46+African PRS variants are denoted by green points. **B** Stacked strip charts reveal that PRS variants are not enriched for high iHS statistics in Great Britain or Nigeria when compared to the rest of the genome. One sample Kolmogorov-Smirnov goodness of fit tests were used to obtain *p*-values (null hypothesis: iHS percentiles are uniformly distributed). **C***PolyGraph* results. For each PRS, *p*-values refer to tests of polygenic adaptation acting over the entire admixture graph. 1KGP population codes are described in the “Methods” section

Using data from the 1000 Genomes Project (1KGP), we compared risk allele frequencies at CaP-associated loci in Europe and SSA. Figure 2a shows that many CaP-associated loci have large allele frequency differences between continents, the largest of which were observed for SNPs at Xq12 (rs5919393 and rs7888856, detected in multi-ancestry and European cohorts [44, 46]) and 19q13.2 (rs61088131 and rs11672691, detected in European cohorts [38, 46]). Allele frequency differences between populations can be caused by neutral processes like genetic drift as well as local adaptation and genetic hitchhiking. Because of this, we tested whether CaP-associated loci are enriched for signatures of natural selection. Integrated haplotype score (iHS) statistics quantify extended haplotype homozygosity, a pattern that arises when selection acts on new mutations (i.e., there is a hard selective sweep). Under a null hypothesis of neutral evolution, disease-associated loci are expected to have iHS percentiles that are uniformly distributed. Few CaP-associated loci have large iHS statistics, and PRS variants have iHS distributions that resemble the rest of the genome (Fig. 2b). Collectively, this indicates that CaP-associated loci are not enriched for signatures of hard selective sweeps (*p*-values ≥ 0.2189, Kolmogorov-Smirnov tests).

Tests of polygenic adaptation for each set of PRS variants were conducted using *Polygraph* [49]. Note that output from *Polygraph* includes a *p*-value for selection on the entire admixture graph as well as selection parameters for each branch (Fig. 2c). Overall, there are negligible signatures of polygenic selection acting on CaP-associated loci: Schumacher *p*-value = 0.252, Conti *p*-value = 0.414, and PHS46+African *p*-value = 0.672. Compared to neutral expectations there appears to have been a decrease in the predicted risk of CaP on the branch leading to Japan (JPT).

Allele frequency differences contribute to how well PRS are able to distinguish between case/control status in different populations and existing PRS are more likely to contain European polymorphisms than African polymorphisms [50]. Because SNP heritability is maximized at intermediate allele frequencies [51], PRS variants in the shaded region of Fig. 2a are more informative about CaP risks in Europe than Africa, assuming equivalent effect sizes in both populations. For each PRS, there is an excess of variants in the shaded region (Schumacher *p*-value = 4.098 × 10^−8^, Conti *p*-value = 1.343 × 10^−6^, PHS46+African *p*-value = 6.575 × 10^−5^, two-sided binomial tests). Note that this novel population genetic approach does not require individual-level phenotype data. Focusing on CaP, PRS variants are more likely to have African allele frequencies that are close to zero or one than European allele frequencies that are close to zero or one (compare the left and right sides of Fig. 2a to the top and bottom). This suggests that SNP ascertainment bias contributes to the limited transferability of PRS between Europeans and other populations [50].

**Fig. 3:**
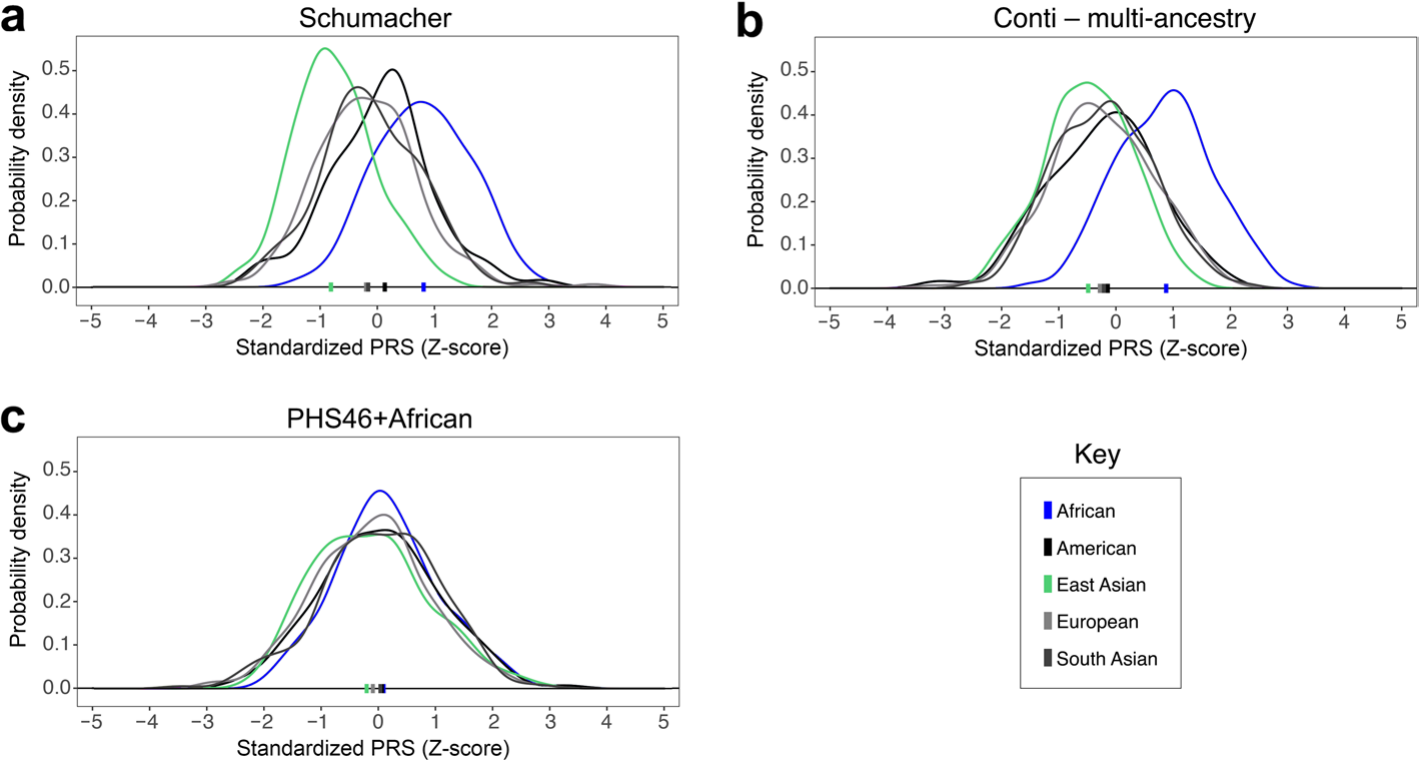
PRS distributions for continental populations from the 1000 Genomes Project. Higher standardized PRS values indicate higher predicted risks of CaP. Colored bars indicate the median PRS for each continental population. Note that admixed African American (ASW) and African Caribbean (ACB) individuals were included in the African group, as opposed to the American group

We examined how predicted risks of CaP vary across the world by applying the Schumacher, Conti, and PHS46+African PRS to 1KGP data (Fig. 3). Recall that these polygenic predictors are nested: the multi-ancestry Conti PRS builds upon the Schumacher PRS, and the PHS46+African PRS builds upon a prior PRS by including three SNPs that were ascertained in men of African descent. Rank orders of continents are consistent with epidemiological data; predicted risks of CaP are highest for Africans and lowest for East Asians, and PRS differences between African genomes and non-African genomes are statistically significant (*p*-values < 2.2 × 10^−16^, Mann-Whitney *U* tests). However, continental differences in risk score distributions are smaller for the PHS46+African PRS than the Schumacher and Conti PRS. This suggests that at least some of the rightward PRS shifts observed for Africans may be due to ascertainment bias. An alternative possibility is that differences in PRS shifts are due to the numbers of variants in each risk score.

### Prostate cancer risk prediction in sub-Saharan Africa: case vs. control status

**Fig. 4:**
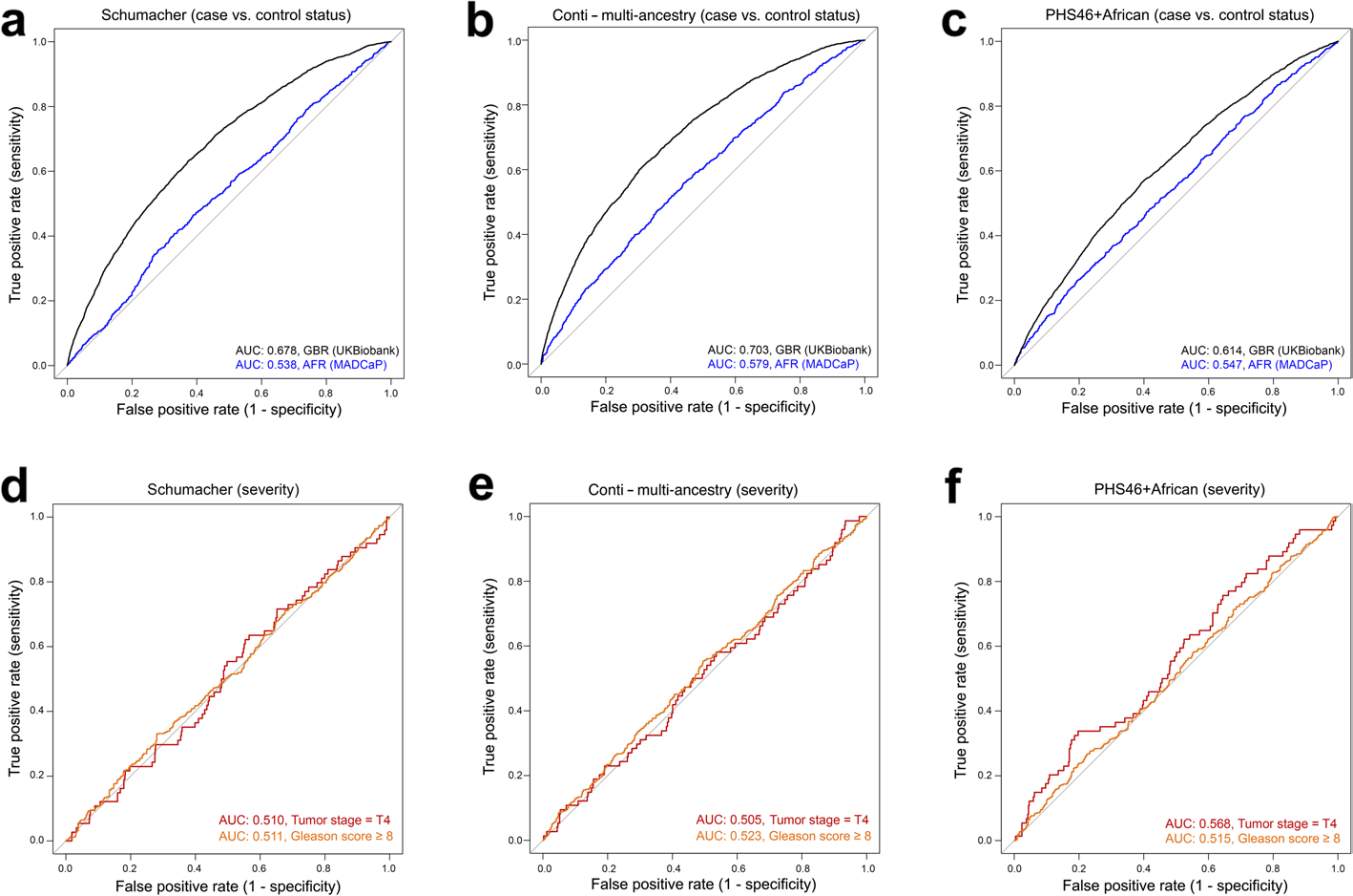
Receiver operator characteristic (ROC) curves for different polygenic risk scores. **A–C** Ability of PRS to distinguish between cases and controls (European and African data). **D–F** Ability of PRS to distinguish between cases that have aggressive and non-aggressive forms of CaP (African data). CaP was classified as aggressive if tumor stage = T4 (opposed to T1, T2, or T3) or Gleason score ≥ 8 (as opposed to Gleason score ≤7), and separate analyses were run for each classifier

Using British samples from the UKBB and SSA samples from the MADCaP Network, we tested how well PRS are able to distinguish between case/control status after correcting for covariates such as age and principal components. Summary statistics of these comparisons can be found in Table 2. Note that proxy variants were used when CaP-associated loci were not directly genotyped and that the relative proportion of proxy variants was larger for MADCaP data than UKBB data (Additional file 1: Table S1). Here we focus on the optimal sets of PRS variants for European and African populations (see “Methods” section for details). Similar results arise if shared sets of PRS variants are used for both continental populations (Additional file 2: Table S2). The receiver operating characteristic (ROC) curves shown in Fig. 4a–c illustrate that predictions of case/control status perform better among men of European descent than among men of African descent. These differences were statistically significant for each PRS. Area under the curve (AUC) statistics for the Schumacher PRS were 0.678 for UKBB samples and 0.538 for MADCaP samples (*p*-value < 2.2 × 10^−16^, DeLong’s test); AUC statistics for the multi-ancestry Conti PRS were 0.703 for UKBB samples and 0.579 for MADCaP samples (*p*-value < 2.2 × 10^−16^, DeLong’s test); and AUC statistics for the PHS46+African PRS were 0.614 for UKBB samples and 0.547 for MADCaP samples (*p*-value < 4.785 × 10^−6^, DeLong’s test).

**Table 2:** Ability of PRS to distinguish between case and control status using the optimal set of variants for European and African datasets. Area under the curve (AUC) statistics and covariate-adjusted odds ratios (OR) are shown for each PRS. These odds ratios involve comparisons between individuals who have a PRS in the top decile to individuals who have a PRS in the middle 20%—i.e., they quantify the how well a risk score is able to distinguish between cases and controls for different parts of a PRS distribution after correcting for age and first 10 principal components

PRS source	PRS ancestry	AUC_UKBB_ (95% CI)	OR_UKBB_ (95% CI)	AUC_MADCaP_ (95% CI)	OR_MADCaP_ (95% CI)
Schumacher	European	0.675 (0.662–0.689)	3.59 (2.89–4.49)	0.538 (0.516–0.56)	1.23 (0.91–1.67)
Conti	Multi-ancestry	0.703 (0.694–0.713)	5.29 (4.26–6.59)	0.579 (0.558–0.601)	1.86 (1.41–2.47)
Conti	European	0.707 (0.698–0.717)	5.71 (4.59–7.14)	0.541 (0.519–0.563)	1.60 (1.20–2.12)
Conti	African	0.671 (0.662–0.681)	4.00 (3.17–4.95)	0.585 (0.563–0.607)	2.01 (1.52–2.67)
Conti	Asian	0.662 (0.652–0.672)	3.32 (2.67–4.14)	0.533 (0.511–0.555)	1.83 (1.38–2.45)
Conti	Hispanic	0.678 (0.668–0.688)	3.93 (3.17–4.91)	0.527 (0.505–0.549)	1.65 (1.24–2.21)
PHS46	European	0.612 (0.598–0.627)	2.37 (1.90–2.96)	0.502 (0.48–0.524)	0.95 (0.70–1.28)
PHS46+African	European + African	0.608 (0.594–0.622)	2.50 (2.00–3.15)	0.547 (0.525–0.569)	1.58 (1.20–2.11)

Odds ratios (OR) can also be used to quantify the effectiveness of PRS. Note that the OR described here do not refer to the relative risks of CaP in Europe and Africa. Instead, they refer to the ability of each PRS to distinguish between case and control status within each continental dataset, after correcting for age and principal components. We calculated covariate-adjusted ORs using generalized linear models, comparing individuals with high risk scores (population-specific PRS percentiles above 90%) to individuals with moderate risk scores (population-specific PRS percentiles between 40% and 60%). In general, European ORs were larger than African ORs (Table 2). This indicates that existing CaP PRS were more effective at distinguishing between cases and controls for European samples. For example, the multi-ancestry weights from the Conti PRS yielded an OR of 5.29 for individuals from the UKBB and an OR of 1.86 for individuals from the MADCaP Network. Collectively, these results reveal that existing PRS are better at distinguishing between case/control status in European populations than African populations.

### Ancestry-matched polygenic predictions of CaP risk

We assessed the impact of applying ancestry-specific weights from the Conti PRS to case and control data from Europe and Africa. For British individuals from the UKBB, multi-ancestry and European PRS weights performed the best (Table 2). Other ancestry-specific PRS weights (African, Asian, and Hispanic) yielded lower AUC scores and odds ratios for British individuals. For individuals from the MADCaP Network, genetic predictions performed best when we used African weights (AUC = 0.585, 95% CI 0.563–0.607; OR = 2.01, 95% CI 1.52–2.67). Other ancestry-specific PRS weights (Asian, European, and Hispanic) yielded lower AUC scores and OR for African individuals (Table 2). Combining MADCaP data from Senegalese, Ghanaian, and Nigerian study sites, we found that African weights from the Conti PRS yielded an AUC of 0.611. By contrast, South African study sites yielded an AUC of 0.560 for the Conti PRS with African weights. These findings reveal that genetic predictions of CaP risk perform better for West African men than South African men (*p*-value = 0.021, DeLong’s test).

We also examined the benefits of including ancestry-matched information in polygenic hazard scores (Table 2). The PHS46 predictor contains genetic variants that were ascertained in men of European descent, and the PHS46+African predictor contains three additional variants that were ascertained in men of African descent. Including these additional variants resulted in improved AUC statistics (0.547 vs. 0.502) and odds ratios (1.58 vs. 0.95) for African individuals from the MADCaP Network. Taken together, these findings indicate that using ancestry-matched or multi-ancestry risk scores improve genetic predictions of cancer risk in Ghana, Nigeria, Senegal, and South Africa.

### Prostate cancer risk prediction in sub-Saharan Africa: disease severity

We also tested how well PRS can distinguish between individuals who have more severe forms of CaP. Here, we focused on two different ways of classifying CaP as aggressive: tumor stages and Gleason scores. Tumor stage data were available for 1002 MADCaP cases and Gleason score data were available for 1068 MADCaP cases. Neither of these clinical phenotypes were available for UKBB samples. We classified CaP as aggressive if tumor stage = T4 (opposed to T1, T2, or T3) or Gleason score ≥ 8 (as opposed to Gleason score ≤7). ROC curves for aggressive CaP are shown in Fig. 4d–f. When risk scores were used to distinguish between individuals with different tumor stages, the Schumacher PRS yielded an AUC statistic of 0.510 (95% CI 0.438–0.578), the Conti PRS yielded an AUC statistic of 0.505 (95% CI 0.435–0.574), and PHS46+African risk score yielded an AUC statistic of 0.568 (95% CI 0.494–0.631). When risk scores were used to distinguish between individuals with different Gleason scores, the Schumacher PRS yielded an AUC statistic of 0.511 (95% CI 0.475–0.547), the Conti PRS yielded an AUC statistic of 0.523 (95% CI 0.488–0.559), and PHS46+African risk score yielded an AUC statistic of 0.515 (95% CI 0.479–0.550). Comparisons of individuals in the top PRS decile to individuals in the middle 20% of each PRS distribution yielded only modest odds ratios. ORs ranged between 0.96 and 1.14 when tumor stages were used to classify CaP as aggressive, and ORs ranged between 1.13 and 1.26 when Gleason scores were used to classify CaP as aggressive (Additional file 3: Table S3). Overall, our findings indicate that polygenic predictors provide only minimal insight into the histopathology of CaP in African men.

## Discussion

Distributions of PRS vary across continental populations. Despite appreciable allele frequency differences between continents, PRS variants are not enriched for signatures of selection acting on new mutations (i.e., hard selective sweeps). This suggests that allele frequency differences at CaP-associated loci are largely driven by genetic drift and other neutral evolutionary processes (e.g., founder effects and population bottlenecks). Allele frequency differences also contribute to the relative effectiveness of PRS in different populations.

Using British data from the UKBB and SSA data from the MADCaP Network, we examined how well genetic predictions of CaP generalize across populations. PRS were much more effective at predicting case vs. control status in men of European descent than in men of African descent. SNP ascertainment bias incurred by using genetic variants discovered in European populations likely contributes to these differences in PRS [26, 31, 50]. In agreement with recent findings [52], our results indicate that ancestry-matched risk scores outperform risk scores that are not ancestry-matched. There is increasing evidence that the generalizability of polygenic predictions drops off in proportion to the genetic distance between populations [53]. Consistent with the major geographic sources of African American DNA [54, 55], inclusion of genetic information from African Americans improved PRS performance more for West Africans than South Africans. Although genetic predictions of CaP risk are improved by using ancestry-matched PRS weights, we note that these improvements do not raise AUC statistics beyond 0.611 for SSA data. Because of this, we caution that existing PRS have only a modest ability to predict CaP risks in African men. Genetic architectures of diseases like CaP can differ between populations [56], and many genetic variants that contribute to risks of CaP in SSA remain undiscovered.

Additional factors may contribute to the observed differences in PRS performance. First, genotype data comes from arrays (i.e., SNP ascertainment bias exists) [26]. Second, imputation accuracy varies across populations and the use of proxy variants can reduce the effectiveness of each PRS [57]. Third, clinical diagnosis of CaP cases can differ across study sites [47]. Fourth, the studies used to generate each PRS have different sample sizes, and this affects the weightings of individual PRS variants [58].

PRS performance was poorer for tumor stage and Gleason score than for case/control status. This finding is not surprising, given the relative paucity of GWAS loci that have been associated with aggressive or early-onset CaP [59]. Importantly, published PRS use germline variants, most of which have European minor allele frequencies that are above 5% (Fig. 2a). Somatic mutations in prostate tissue also contribute to cancer risk [60], but their effects are generally not included in PRS calculations. Because of this, the relatively low AUC statistics and ORs shown in Additional file 3: Table S3 suggest that rare germline variants and/or somatic mutations may be important drivers of CaP aggressiveness.

## Conclusions

Here, we found that genetic predictions of CaP risks perform poorly if the study sample does not match the ancestry of the original GWAS. In a clinical setting, predictions are likely to benefit from the inclusion of additional factors (e.g., family history, age, and PSA levels). Going forward, transferability of genetic risk scores can be improved by incorporating evolutionary [50] as well as linkage disequilibrium [61] information to better infer effect sizes of risk alleles in understudied populations. Unless well-powered GWAS are undertaken in diverse populations, the accuracy and utility of PRS will be sub-optimal, exacerbating disparities in risk prediction and subsequent disease management [62].

## Methods

### Population genetic datasets

We extracted genotype and phenotype data for 191,941 British males of European descent from the UKBB [63, 64] (3049 CaP cases and 188,892 controls, self-reported code 1044 in data field 20001). African men aged 40 years or older were recruited in a multicenter, hospital-based case-control study from seven MADCaP sites between 2016 and 2019 [47]: the Hôpital Général de Grand Yoff/Institut de Formation et de Recherche en Urologie in Dakar, Senegal (HOGGY); 37 Military Hospital in Accra, Ghana (37 Military); Korle-Bu Teaching Hospital in Accra, Ghana (KBTH); University College Hospital in Ibadan, Nigeria (UCH); University of Abuja Teaching Hospital in Abuja, Nigeria (UATH); Wits Health Consortium/National Health Laboratory Services in Johannesburg, South Africa (NHLS/WITS); and Stellenbosch University in Cape Town, South Africa (SU). Many African cases first present with symptoms, which may account for the high proportions of aggressive CaP shown in Table 1. CaP cases and controls were frequency matched by age and study site. African individuals were genotyped using the MADCaP Array, a custom genotyping platform optimized for detecting genetic associations with prostate cancer in sub-Saharan African populations [48]. Details about sample accrual can be found in Andrews et al. [47], and details about SNP calling and QC filtering be found in Harlemon et al. [48]. MADCaP samples were excluded if marker missingness exceeded 5%. A total of 2631 MADCaP samples were analyzed in downstream analyses (1298 CaP cases and 1333 controls, Table 1). Two-dimensional MDS and ADMIXTURE [65] plots were used to visualize the population structure of MADCaP samples (optimal *K* = 3, as per [48]). Self-reported British cases and controls from the UKBB cohort were analyzed. We excluded UKBB individuals who were outliers in PCA space (i.e., all UKBB individuals were required to be within two standard deviations of the mean for both of the first two principal components). To avoid artifacts, UKBB data were randomly downsampled to yield similar ratios of cases to controls as MADCaP Network data. After filtering, this yielded 5387 samples from the UKBB (2700 CaP cases and 2687 controls).

### Polygenic risk score (PRS) calculations

PRS were generated using sets of CaP-associated loci as per Schumacher et al. [38], Conti et al. [44], and Karunamuni et al. [46]. Proxy SNPs were imputed for PRS variants that were not directly genotyped in the UKBB and MADCaP Network datasets using the LDproxy function of LDlink [66] to identify genotyped SNPs in linkage disequilibrium with PRS variants. PRS variants that lacked proxies (*r*^2^ < 0.4) were excluded. The indel rs11293876 is absent from dbSNP, causing the Schumacher PRS to shrink to a total of 146 markers. As per [67], genotypes at rs72725854 were inferred using a pair of closely linked markers (rs114798100 and rs1119069), as opposed to a single proxy, causing the Conti PRS to expand to a total of 270 markers. Details about PRS variants and proxies are listed in Additional file 1: Table S1. Note that the ideal proxy for one continental dataset need not be the ideal proxy for another continental dataset. Two different approaches were used to obtain PRS variants. First, we obtained the optimal set of PRS variants for each continental dataset (i.e., the best set of predictors for Europe and Africa). Second, we obtained a shared set of PRS variants for both continental populations (i.e., an identical set of variants for both datasets). Focusing on optimal sets of PRS variants for Europe and Africa, UKBB genotype data were available for 93% of Schumacher variants, 91% of Conti variants, and 91% of PHS46+African variants (including proxies). Similarly, MADCaP genotype data were available for 94% of Schumacher variants, 83% of Conti variants, and 98% of PHS46+African variants (including proxies). Focusing on shared variants found in both continental datasets: genotype data were available for 89% of Schumacher variants, 82% of Conti variants, and 86% of PHS46+African variants (including proxies). All original PRS variants were used when risk scores were calculated for males from the 1KGP.

Standard approaches were used to generate PRS for each individual [68]. For each PRS variant, risk alleles were counted for each individual; i.e., the allele dose at locus *i* in individual *j* (*d*_*i,j*_) ranges from 0 to 2. Mean counts of risk alleles for each study site were used to fill any missing genotype data. This was done to avoid biases whereby individuals with more missing data have lower polygenic scores. In practice, missing data had little effect, as overall missingness rates of PRS variants were low for each sample (0.67% on average). For each risk score, allele doses were weighted using adjusted effect sizes: $\beta_i=\ln\left({\mathrm{OR}}_{\mathrm i}\right)\times r_i^2$ (where ${r}_i^2$ indicates how well proxy SNPs tag PRS variants). PRS were generated for each individual by summing across L loci: ${\mathrm{PRS}}_j=\sum_{i=1}^{\mathrm{L}}{d}_{i,j}{\beta}_i$. As per [50], raw risk scores were converted to a standardized scale across all samples (mean of 0 and a standard deviation of 1). PRS were calculated for 1233 males from phase 3 of the 1KGP [69], 5387 British males from the UKBB, and 2631 African males from the MADCaP Network. Note that the Conti PRS contains ancestry-specific weights (i.e., different effect sizes for individuals of European, African, Asian, and Hispanic descent), as well as multi-ancestry PRS weights. Additional details about these weights can be found in Supplementary Table S4 of [44].

### Scans of selection

Integrated haplotype scores (iHS) quantify signatures of recent natural selection [70]. PRS variants from the Conti, Schumacher, and PHS46+African PRS were merged with hapbin [71] iHS data from Great Britain (GBR) and Nigeria (YRI). iHS statistics were available for autosomal SNPs with minor allele frequencies > 0.05. To test whether PRS variants were enriched for signatures of selection, we compared iHS statistics at CaP-associated loci to genome-wide distributions of iHS statistics.

Signals of polygenic adaptation for sets of CaP-associated loci were also tested using *PolyGraph* [49]. *PolyGraph* infers branch-specific selection parameters on admixture graphs using a Markov Chain Monte Carlo (MCMC) algorithm. Data requirements of *PolyGraph* are summary statistics from GWAS for a trait, a set of neutral SNPs, ancestral state information, and an admixture graph of the populations being studied. *SNPSnap* [72] was used to obtain frequency-matched neutral SNPs. *MixMapper* [73] was used to build the admixture graph. Phase 3 data from the 1KGP [69] was used as a reference for building admixture graphs. 1KGP population codes are as follows: British in England and Scotland (GBR), Iberian in Spain (IBS), Yoruba in Nigeria (YRI), Mende in Sierra Leone (MSL), Bengali from Bangladesh (BEB), Sri Lankan Tamil (STU), Han Chinese in Beijing (CHB), Japanese in Tokyo (JPT), and Peruvian from Lima (PEL).

### Tumor stages and Gleason scores

Standardized procedures were used to collect clinical data on CaP and quantify the aggressiveness of CaP in MADCaP samples [47]. Clinical tumor stages refer to whether cancers are restricted to the prostate gland [74], and biopsy Gleason scores indicate whether biopsies reveal abnormal histology patterns [75]. Using recently published guidelines [76], we classified CaP as aggressive if tumor stage = T4 or Gleason score ≥ 8. Analyses were run separately for tumor stage and Gleason score classifiers. Tumor stage and Gleason score data were available for 1002 and 1068 MADCaP CaP cases, respectively. Tumor stage and Gleason score data were not available for UKBB cases.

### Statistical analyses

Two-sided binomial tests were used to infer whether European or SSA allele frequencies are closer to 0.5 (note that SNP heritabilities are maximized at intermediate allele frequencies [35]). This novel approach involved comparing counts of SNPs in the shaded bow-tie region of Fig. 2a to counts of SNPs lying outside the shaded region. PRS distributions for continental populations were compared using Mann-Whitney *U* tests. Using R, one-sample Kolmogorov-Smirnov goodness of fit tests were used to infer whether iHS percentiles of PRS variants are uniformly distributed. Sets of frequency-matched SNPs were used to infer *p*-values via *PolyGraph* [49]. ROC curves and AUC statistics were used to quantify how well PRS predict case/control status and CaP aggressiveness using logistic regression. Perfect classifiers have AUC statistics of 1, and classifiers that are no better than chance have AUC statistics of 0.5. The pROC package in R was used to calculate 95% confidence intervals for AUC statistics, and DeLong’s test was used to test whether differences in AUC statistics were statistically significant [77]. For each PRS and population combination, odds ratios were calculated using covariate-adjusted generalized linear models in R. Covariates used were age and the first 10 principal components for each continental dataset. Median values were used when age covariates were missing. All odds ratio calculations were population-specific (i.e., they focused on either the PRS distributions of UKBB or the PRS distributions of MADCaP samples, rather than a pooled PRS distribution).

## Supplementary Information

Additional file 1: Table S1. Details about PRS variants and proxies used in this paper.

Additional file 2: Table S2. Ability of PRS to distinguish between case and control status using a shared set of variants for both continental datasets.

Additional file 3: Table S3. Ability of PRS to distinguish between aggressive and non-aggressive forms of CaP using the optimal set of variants for European and African datasets.

Additional file 4. Peer review history.

## Data Availability

The data underlying this article are available from the MADCaP Data Access Approvals Committee (https://www.madcapnetwork.org/) on reasonable request. Genetic data are also available via dbGaP (accession number: phs002718.v1.p1) [78].
