# Supplementary material for: Testing the generalizability of ancestry-specific polygenic risk scores to predict prostate cancer in sub-Saharan Africa
Source: Genome Biol. 2022 Sep 13;23:194. doi: 10.1186/s13059-022-02766-z (PMC9472407; doi:10.1186/s13059-022-02766-z)
Supplement: Supplementary file 2 — Additional file 2: Table S2. Ability of PRS to distinguish between case and control status using a shared set of variants for both continental datasets. [file 13059_2022_2766_MOESM2_ESM.docx]

**Additional file 2: Table S2**

| PRS source | PRS ancestry | AUC_UKBB_  (95% CI) | OR_UKBB_  (95% CI) | AUC_MADCaP_  (95% CI) | OR_MADCaP_  (95% CI) |
| --- | --- | --- | --- | --- | --- |
| Schumacher | European | 0.68  (0.67 – 0.69) | 4.05  (3.26 - 5.04) | 0.54  (0.51 – 0.56) | 1.40  (1.04 - 1.89) |
| Conti | Multi-ancestry | 069  (0.68 – 0.71) | 3.45  (2.77 - 4.31) | 0.55  (0.53 – 0.57) | 1.05  (0.79 - 1.41) |
| Conti | European | 0.69  (0.68 – 0.71) | 3.52  (2.83 - 4.40) | 0.54  (0.52– 0.57) | 1.14  (0.83 - 1.49) |
| Conti | African | 0.67  (0.65 – 0.68) | 2.90  (2.33 - 3.62) | 0.55  (0.53 – 0.58) | 1.47  (1.10 - 1.96) |
| Conti | Asian | 0.66  (0.64 – 0.67) | 2.76  (2.21 - 3.45) | 0.55  (0.53 – 0.57) | 1.06  (0.79 - 1.42) |
| Conti | Hispanic | 0.67  (0.65 – 0.68) | 2.67  (2.14 - 3.33) | 0.53  (0.51– 0.55) | 1.26  (0.94 - 1.69) |
| PHS46 | European | 0.63  (0.62 – 0.65) | 2.20  (1.75 - 2.76) | 0.51  (0.49 – 0.53) | 0.79  (0.58 - 1.07) |
| PHS46+African | European + African | 0.63  (0.61 – 0.64) | 2.20  (1.75 - 2.76) | 0.51  (0.49 – 0.54) | 0.86  (0.64 - 1.17) |

**Table S2.** Ability of PRS to distinguish between case and control status using a shared set of variants for both continental datasets. Area under the curve (AUC) statistics and covariate-adjusted odds ratios (OR) are shown for each PRS. These odds ratios involve comparisons between individuals who have a PRS in the top decile to individuals who have a PRS in the middle 20% of each PRS distribution after correcting for age and the first 10 principal components.
