# Supplementary material for: Testing the generalizability of ancestry-specific polygenic risk scores to predict prostate cancer in sub-Saharan Africa
Source: Genome Biol. 2022 Sep 13;23:194. doi: 10.1186/s13059-022-02766-z (PMC9472407; doi:10.1186/s13059-022-02766-z)
Supplement: Supplementary file 3 — Additional file 3: Table S3. Ability of PRS to distinguish between aggressive and non-aggressive forms of CaP using the optimal set of variants for European and African datasets. [file 13059_2022_2766_MOESM3_ESM.docx]

**Additional file 3: Table S3**

| Aggressiveness criterion | PRS source | PRS ancestry | AUC_MADCaP_  (95% CI) | OR_MADCaP_  (95% CI) |
| --- | --- | --- | --- | --- |
| Tumor stage = T4 | Schumacher | European | 0.510  (0.438 – 0.578) | 1.14  (0.93-1.40) |
| Tumor stage = T4 | Conti | Multi-ancestry | 0.505  (0.435 – 0.574) | 1.17  (0.95 - 1.43) |
| Tumor stage = T4 | PHS46+African | European + African | 0.568  (0.494 – 0.631) | 0.96  (0.78-1.17) |
| Gleason Score ≥ 8 | Schumacher | European | 0.511  (0.475 – 0.547) | 1.13  (1.02 - 1.25) |
| Gleason Score ≥ 8 | Conti | Multi-ancestry | 0.523  (0.488 – 0.559) | 1.26  (1.15 - 1.40) |
| Gleason Score ≥ 8 | PHS46+African | European + African | 0.515  (0.479 – 0.550) | 1.18  (1.07 - 1.130) |

**Table S3.** Ability of PRS to distinguish between aggressive and non-aggressive forms of CaP using the optimal set of variants for European and African datasets. CaP was classified as aggressive if tumor stage = T4 (opposed to T1, T2 or T3) or Gleason score ≥ 8 (as opposed to Gleason score ≤7), and separate analyses were run for each classifier. Area under the curve (AUC) statistics and covariate-adjusted odds ratios (OR) are shown for each PRS. These odds ratios involve comparisons between individuals who have a PRS in the top decile to individuals who have a PRS in the middle 20% of each PRS distribution after correcting for age and the first 10 principal components.
