# Supplementary material for: Testing the generalizability of ancestry-specific polygenic risk scores to predict prostate cancer in sub-Saharan Africa
Source: Genome Biol. 2022 Sep 13;23:194. doi: 10.1186/s13059-022-02766-z (PMC9472407; doi:10.1186/s13059-022-02766-z)
Supplement: Supplementary file 4 — Additional file 4. Peer review history. [file 13059_2022_2766_MOESM4_ESM.docx]

**Review history**

**First round of review**

**Reviewer 1**

Comments on the manuscript "Testing the generalizability of ancestry-specific polygenic risk scores to predict prostate cancer in sub-Saharan Africa"

This paper compares existing prostate cancer PRS in African populations and the UK Biobank. The PRS perform less well in the African populations, suggesting limited transferability. The topic is very timely and interesting, but I have a number of comments that would improve the manuscript.

1. Indicate in the abstract which PRS were used.

2. Could the large differences in AUC be due to differences in case diagnosis and ascertainment?

3. Instead of just giving the AUCs in the abstract, I would also include the ORs comparing top decile to middle.

Introduction

4. "Most genotyping arrays use markers ascertained in non-African populations"

Is this true with the current genotyping arrays? References are more to the PRS being developed in EUR populations.

5. Clarify that the Schumacher, Conti, and PHS46+African PRS are nested and cover the same variants. In fact, it's probably not necessary to present all three of these since they are so highly correlated (Conti is the most recent, and the multiancestry PRS should be used for the main comparison). What would be more interesting is seeing what happens when additional information is incorporated into the development or weighting of the PRS (e.g., using PRS-csx).

Results

6. Indicate the chromosomal regions where rs5919393, rs61088131, rs7888856, and rs11672691 are located, and what discovery population they were originally detected in.

7. "Allele frequency differences contribute to how well PRS are able to distinguish between case/control status in different populations."

Is this a general statement or specific to these results? If the former, please include a reference. If the latter, this statement seems premature since the results on performance are not yet shown. What this paragraph of results do show is that the PCa PRS SNPs have lower variability in the African population.

8. "Note that this novel population genetic approach does not require individual-level phenotype data to infer the relative performance of PRS in different populations."

Ditto above. This paragraph does not present any results regarding PRS performance.

9. "However, continental differences in risk score distributions are smaller for the PHS46+African PRS than the Schumacher and Conti PRS. This suggests that at least some of the rightward PRS shifts observed for Africans may be due to ascertainment bias."

Couldn't this simply reflect the smaller number of variants included in the PHS46+African PRS, rather than an ascertainment bias?

10. The ORs are from comparing the top to bottom deciles. For comparison's sake with other publications, the authors should contrast the 90th percentile to the middle two deciles.

11. "For example, the Schumacher PRS yielded an OR of 9.83 for individuals from the UKBB and an OR of 1.36 for individuals from the MADCaP Network."

Why select one of the most extreme comparisons, using the PRS that was not developed to be used for multiancestries? The results from the Conti multiancestry should be presented (and this should be the focus of the paper).

12. "Taken together, these findings indicate the using ancestry-matched or multi-ancestry risk scores improve genetic predictions of cancer risk."

This has been shown already in Conti. I think the point maybe is that for African populations…?

13. Why do the disease severity AUC results for different PRS approaches vary depending on whether tumor stage and Gleason score are combined versus just Gleason score alone?

Discussion

14. "Predicted risks of CaP vary across continental populations."

Do you mean that the distribution of PRS vary across populations?

15. "Somatic mutations also contribute to cancer risk"

Do you mean rare or de novo germline mutations (as opposed to somatic mutations in tumor tissue?). If you mean somatic in prostate tissue, how would these be measured for inclusion in a PRS?

16. "In a clinical setting, predictions are likely to benefit from the inclusion of additional factors (e.g., family history, age, somatic mutations, and PSA levels)."

This is the first time other factors are mentioned. Did the authors consider incorporating them into their analyses? Also, see above question regarding what is meant by 'somatic mutations'.

17. "Going forward, transferability of genetic risk scores can be improved by incorporating evolutionary [46] as well as linkage disequilibrium [53] information to better infer effect sizes of risk alleles in understudied populations."

Why not do this here to try and improve the PRS?

Methods

18. 40% of the AFR cases have Gleason >=8. This is substantially higher than that observed in EUR populations. One possible explanation is differences in case ascertainment in Africa (e.g., non-PSA screened). What impact would this have on using a PRS developed in screened EUR populations? Is the issue with how much screening has been done, not as much ancestry? What are the implications for using the PRS to detect clinically meaningful cancer?

19. "UKBB data were randomly down-sampled to yield the same numbers of cases and controls as MADCaP Network data."

Why down sample the UKB data? To compare the stability of the results (e.g., 95% CIs)? Whatever the reason, please justify.

20. There were 8% fewer SNPs from the Conti paper used in the AFR PRS than EUR PRS. What happens when the same variants are used for both analyses? Are any of the missing SNPs specific to or with higher frequencies in the AFR population?

21. "Mean counts of risk alleles for each study site were used to fill any missing genotype data"

What was the rate of missingness among the different populations?

22. "Two-sided binomial tests were used to infer if SNPs contribute more to SNP heritability in Europe than in SSA (i.e., whether European or SSA allele frequencies are closer to 0.5)."

MAF is not the only contributor to SNP heritability. This is really just a comparison of whether the allele frequencies are closer to 0.5, and should be stated as such.

23. The odds ratios should be calculated from logistic regression, not from the counts of cases and controls. The regression models should adjust for age and other covariates (including principal components for ancestry).

**Reviewer 2**

The manuscript by Kim et al. is an important investigation how reliable are polygenic risk scores (PRS), which are still largely biased towards European-decedent data, to predict risk of prostate cancer and of aggressive forms of prostate cancer in males of African ancestry. They demonstrate how incorporation of ancestry-informed markers in the PRS do improve accuracy of inferences (not so much for the aggressive form), but, nonetheless, how far we still are of having identifying essential risk markers for the African ancestry. It is not surprising that PRS inferences within African work better for those of western African background, given that most information we have are for African-Americans, whom are mainly of western African ancestry, or from MADCaP excess of samples from western Africa. I agree that the publication of this manuscript is currently important, and raises awareness of how important it is to fund international collaboration with teams across Africa, as the MADCaP example.
I have the following suggestions that can help improving the manuscript:
1- My main concern is about the evaluation of selection on the rs5919393, rs61088131, rs7888856, and rs11672691 markers. As the authors have demonstrated, the phenotypes under evaluation are under the effect of the interaction between many genes - if there is some selection effect, it is probably polygenic selection. So, it is highly improbable that those 4 markers were under the effect of a strong selective sweep. To apply just one algorithm that is only able to detect strong selective sweep is currently quite limiting. Check, for example, the calculation of "polygenic selection of complex traits" methods section of the manuscript by Lopez et al. 2019. Current Biology 29: 2926-2935.

2- Another important aspect that the authors should be more careful in explaining and evaluating is the imputation of the genotypes of the PRSs. Imputation is not so reliable for African populations, given the huge bias in complete genome sequences between European and African individuals. What was the ratio of markers that needed imputation between European- and African-associated CaP markers? This is an important information for the Results section, and should not be left only for the Material and Methods section.

3- Results section, page 9, after authors describe the "case/control ratios among individuals with high risk scores (population-specific PRS percentiles above 90%) to case/control ratios among individuals with low risk scores (population-specific PRS percentiles below 10%)", I got lost when they say "the Schumacher PRS yielded an OR of 9.83 for individuals from the UKBB and an OR of 1.36 for individuals from the MADCaP Network" that these values would mean (individual) ORs or those ratios. I think that another nomenclature that not simply OR should be used here, for clarification purposes. I immediately think about the OR at individual level - this is even worse if only looking at Table 2. The message it transmits is totally wrong - risk of CaP in Europeans would be huge compared to Africans.

4- Results section, page 9, please give more information on the "ancestry-specific weights". What are these? I did not find any information even in Material and Methods. But definitely, it should be included in both sections. I had to go to Supplementary Table to check this - I understood that the weighing is by using different beta calculated in various ancestry groups of Conti dataset. Clearly state so, and provide information on sample sizes for each group.

5- In Material and Methods, Page 14/15, what do you really mean when you say "Mean counts of risk alleles for each study site were used to fill any missing genotype data"? If I am understanding correctly, when a genotype for a marker is missing for one individual, your estimate the mean frequency in the population for the risk allele and include that information instead of considering it a missing genotype? I do not agree with this. This is even worse for markers relevant for African ancestry-associated risk, as we do not have so much information about allele frequencies. The authors should at least state how much missing information did they allow for the individuals. If they did not include any restriction, maybe they have individuals for whom the PRS was based on 80%, 50%, 40% (???) inferred genotypes. How biasing was this additional factor in their analyses? Remember that this adds up to the fact that many genotypes had to be input.

6- In Figure 2, further information is needed for the "American" 1000Genomes group. Does it include "African ancestry in SW USA - ASW" and "African Caribbean in Barbados - ACB"? If yes, it does not make sense.

**Reviewer 3**

This study applies polygenic risk scores to a case control data set from sub-Saharan Africa to see whether performance differs within the African dataset from performance within a comparably sized random sample of European data drawn from the UK biobank. The question of performance in diverse data set is an important one, especially for prostate cancer, where prior work has shown the potential clinical utility of predicted genetic risk to guide screening decisions. The dataset used here is particularly interesting because it is drawn directly from several areas of Africa. The authors are to be commended for collecting this African data set and for considering generalization performance from several angles.

1. It would be helpful to have any available additional detail about the characteristics of the individuals including in this study. For example, how much admixture is observed in these populations? South Africa, for example, is known to have multiple ethnicities. Can a brief introduction be given for the populations included from each site?
2. The finding that odds ratios in MADCaP are much smaller than in UK bio bank is important. Indeed, the difference in performance appears much larger between these two data sets than was reported previously for prostate cancer (PMID: 33792693, PMID: 33623038). The conclusion for the present study is written, "genetic prediction of prostate cancer risks perform poorly if the study sample does not match the ancestry of the original GWAS." It appears, however, that sometimes scores will perform relatively better or worse out of the original sample. This is both important to know and a suggestion that there is work to be done to understand what drives this variance in performance and how to predict it. The discussion and conclusion would be more informative with this additional context.
3. If available, more information should be given about how these cases were obtained. Is there commonly any early detection with PSA testing, or are these generally patient presenting with suspicious symptoms? The age range of the included individuals is fairly advanced, which could be noted in the text.
4. For completeness, please add confidence intervals for the disease severity analyses.
5. (Minor) Any thoughts as to why the continental differences in score distributions were smaller for the 46-variant score? Is it possible that variants with smaller effect sizes are more likely to be differentially distributed across populations?
6. (Minor) The last paragraph on page 9 (lines 49 through 55) mentions that European versus multi ancestry weights performs better for AUC or odds ratio, respectively. This is of unclear significance. Confidence intervals should be reported in the text, at least, even if no further explanation can be given.

**Authors’ response to reviewers**

We thank the editors of Genome Biology and three anonymous reviewers for their helpful and thorough comments. Following their suggestions we have multiple improvements to our manuscript, including:

• Additional details about MADCaP Network study populations (plus a new figure)
• Further tests of polygenic adaptation (PolyGraph analyses were conducted by Ujani Hazra, now listed a coauthor
• Risk score analyses involving a shared set of prostate cancer SNPs, as opposed to different optimal sets for Europe and Africa (summarized in a new supplementary table)
• Recalculated odds ratios for polygenic risk scores (the top decile is of risk score distributions is now compared the middle 20%)
• Additional corrections and reviewer-suggested changes

Responses to specific reviewer comments are listed below.


Reviewer #1: Comments on the manuscript "Testing the generalizability of ancestry-specific polygenic risk scores to predict prostate cancer in sub-Saharan Africa"

This paper compares existing prostate cancer PRS in African populations and the UK Biobank. The PRS perform less well in the African populations, suggesting limited transferability. The topic is very timely and interesting, but I have a number of comments that would improve the manuscript.

1. Indicate in the abstract which PRS were used.

Done.


2. Could the large differences in AUC be due to differences in case diagnosis and ascertainment?

Yes, this could be one cause. The revised Discussion section of our manuscript now includes additional details about possible differences in the effectiveness of polygenic predictions. We note that there has been a concerted effort by clinicians in the MADCaP Network to adopt standardized diagnosis procedures. However, a substantial proportion of African CaP cases are only identified after the disease has progressed to late stages. Differences in discovery sample sizes and ancestries for each PRS also contribute to differences in PRS performance.


3. Instead of just giving the AUCs in the abstract, I would also include the ORs comparing top decile to middle.

Done. We now include odds ratios comparing the top 10% to the middle 20% for UKBB and MADCaP data in the abstract and main text of our manuscript. Table 2 has also been modifed to reflect this change.


4. "Most genotyping arrays use markers ascertained in non-African populations". Is this true with the current genotyping arrays? References are more to the PRS being developed in EUR populations.

Yes, most previously published prostate cancer studies use arrays with markers that were largely ascertained in non-African populations. For example, the Illumina Omni 2.5M array was developed from 1000 Genomes Project sequence data (most of the populations of which are non-African), leading to enrichment of polymorphisms that “survived” the out-of-Africa migration. We have slightly reworded this part of the Introduction, and now include additional references related to genotyping arrays. PRS-related references have been moved to the subsequent sentence about the limited generalizability of genetic predictions.


5. Clarify that the Schumacher, Conti, and PHS46+African PRS are nested and cover the same variants. In fact, it's probably not necessary to present all three of these since they are so highly correlated (Conti is the most recent, and the multiancestry PRS should be used for the main comparison). What would be more interesting is seeing what happens when additional information is incorporated into the development or weighting of the PRS (e.g., using PRS-csx).

We have now mention the nested nature of these PRS in the Introduction and Results section of our manuscript. We are intrigued as well about the potential for PRS-csx to yield improved cross-ancestry performance and look forward to future CaP papers using this approach.


6. Indicate the chromosomal regions where rs5919393, rs61088131, rs7888856, and rs11672691 are located, and what discovery population they were originally detected in.

Chromosomal regions and discovery populations are now included in the text of our manuscript.
rs5919393 at Xq12 was detected in a multi-ancestry cohort (Conti et al.)
rs7888856 at Xq12 was detected in a European cohort (Karunamuni et al.).
rs61088131 at 19q13.2; was detected in a European cohort (Schumacher et al.).
rs11672691 at 19q13.2 in a European cohort (Karunamuni et al)
Both Xq12 SNPs are in high LD (R2 = 0.9843 for pooled 1KGP populations), and both 19q13.2 SNPs are in moderate LD (R2 = 0.14083 for pooled 1KGP populations).
Upon further examination we realized that our original joint SFS plot depicted effect allele frequencies rather than risk-increasing allele frequencies. Because some effect alleles in the PHS46+African PRS have negative weights we repolarized allele frequencies when needed - generating a new joint SFS plot. Note that binomial tests of European and African MAFs are unaffected by this repolarization (individual points remained in either the shaded or unshaded regions of the joint SFS plot).


7. "Allele frequency differences contribute to how well PRS are able to distinguish between
case/control status in different populations.” Is this a general statement or specific to these results? If the former, please include a reference. If the latter, this statement seems premature since the results on performance are not yet shown. What this paragraph of results do show is that the PCa PRS SNPs have lower variability in the African population.

It is a general statement, and we now include an reference. To increase clarity we have also removed the subsequent sentence and are more explicit about when we are talking about CaP variants later in the same paragraph.


8. "Note that this novel population genetic approach does not require individual-level phenotype data to infer the relative performance of PRS in different populations." Ditto above. This paragraph does not present any results regarding PRS performance.

In hindsight, this point belongs more in the Discussion section, and we have moved it there (albeit in a watered-down form). The point that we would like to make is that allele frequency information can yield insight about the relative efficacy of genetic predictions in different populations.


9. "However, continental differences in risk score distributions are smaller for the PHS46+African PRS than the Schumacher and Conti PRS. This suggests that at least some of the rightward PRS shifts observed for Africans may be due to ascertainment bias." Couldn't this simply reflect the smaller number of variants included in the PHS46+African PRS, rather than an ascertainment bias?

Potentially yes, but the PRS shifts for the PHS46+African PRS are smaller than what would be expected if shifts were just a function of the number of variants in each PRS. Regardless, we now mention that an alternative explanation involves the different numbers of variants in each PRS.


10. The ORs are from comparing the top to bottom deciles. For comparison's sake with other publications, the authors should contrast the 90th percentile to the middle two deciles.

Done. The revised version of Table 2 now includes these details.


11. "For example, the Schumacher PRS yielded an OR of 9.83 for individuals from the UKBB and an OR of 1.36 for individuals from the MADCaP Network." Why select one of the most extreme comparisons, using the PRS that was not developed to be used for multiancestries? The results from the Conti multiancestry should be presented (and this should be the focus of the paper).

We now focus on the OR from the Conti PRS in this part of the Results section.


12. "Taken together, these findings indicate the using ancestry-matched or multi-ancestry risk scores improve genetic predictions of cancer risk." This has been shown already in Conti. I think the point maybe is that for African populations…?

Conti et al. used an African replication sample from Uganda. By contrast, MADCaP study sites are from Ghana, Nigeria, Senegal, South Africa. We have slightly modified this part of the Results to include the specific countries analyzed in our manuscript.


13. Why do the disease severity AUC results for different PRS approaches vary depending on whether tumor stage and Gleason score are combined versus just Gleason score alone?

Some clarification is needed: we examined AUC results for either tumor stage or Gleason score (as opposed to combining both aspects of histopathology). The Methods section and figure legend have been modified to make this a little more clear.


14. "Predicted risks of CaP vary across continental populations.” Do you mean that the distribution of PRS vary across populations?

Yes, that is correct. We have reworded this sentence for clarity.


15. "Somatic mutations also contribute to cancer risk" Do you mean rare or de novo germline mutations (as opposed to somatic mutations in tumor tissue?). If you mean somatic in prostate tissue, how would these be measured for inclusion in a PRS?

The above statement refers to the latter (somatic mutations in prostate tumors). There has been internal debate among members of the MADCaP Network about why aggressive prostate cancer is so hard to predict using genetic information. Some members of our team lean towards the importance of rare segregating germline variants while others lean towards a somatic tumor mutations (it’s worth noting that many genes that are hotspots for tumor mutations in TCGA aren’t cancer hotspots in the GWAS catalog). Ultimately, prediction of aggressive tumors may need to use a hybrid approach which combines PRS information with other approaches like CancerSEEK (which leverages ctDNA and circulating protein biomarkers).


16. "In a clinical setting, predictions are likely to benefit from the inclusion of additional factors (e.g., family history, age, somatic mutations, and PSA levels)." This is the first time other factors are mentioned. Did the authors consider incorporating them into their analyses? Also, see above question regarding what is meant by 'somatic mutations'.

This is good question, but it lies beyond the scope of this present manuscript. How best to combine multiple risk factors into a single metric remains an open question. We (and others) are definitely interested in exploring this space further in the future. Note that our manuscript focused solely on the ability of genetic information to predict case/control status and aggressive disease. This was a conscious choice, as we wanted to avoid overstating the effectiveness of polygenic risk scores (which some studies inadvertently do by combining genetic information with other factors to generate AUC statistics).


17. "Going forward, transferability of genetic risk scores can be improved by incorporating evolutionary [46] as well as linkage disequilibrium [53] information to better infer effect sizes of risk alleles in understudied populations." Why not do this here to try and improve the PRS?

This is a good idea, but it is beyond the scope of our present manuscript. We are currently conducting a MADCaP GWAS of ~7,500 sub-Saharan African men and are generating an African PRS as part of this ongoing work. An additional project includes identifying the molecular and evolutionary genetic signatures of associations that “travel well” across different continental populations.


18. 40% of the AFR cases have Gleason >=8. This is substantially higher than that observed in EUR populations. One possible explanation is differences in case ascertainment in Africa (e.g., non-PSA screened). What impact would this have on using a PRS developed in screened EUR populations? Is the issue with how much screening has been done, not as much ancestry? What are the implications for using the PRS to detect clinically meaningful cancer?

Interesting observation. The relatively large proportion of African cases could indeed be due to differences in screening. This is something that the Clinical Working Group of the MADCaP Network has been examining. Relevant abstract from the 2021 meeting of the American Urological Association: https://www.auajournals.org/doi/abs/10.1097/JU.0000000000002028.18. Note that although PSA screening is available at all MADCaP sites, it is mainly used for diagnostic purposes, as opposed to screening of healthy patients. Where PRS may be of use is the identification of individuals who would benefit the most from (additional) PSA screening.


19. "UKBB data were randomly down-sampled to yield the same numbers of cases and controls as MADCaP Network data." Why down sample the UKB data? To compare the stability of the results (e.g., 95% CIs)? Whatever the reason, please justify.

Yes, the down-sampling was done to avoid the potential problem of differential ability to estimate AUC statistics and OR in different populations (including 95% CI). In any case, the text of our manuscript has been revised to justify the decision to down-sample individuals from the UKBB.


20. There were 8% fewer SNPs from the Conti paper used in the AFR PRS than EUR PRS. What happens when the same variants are used for both analyses? Are any of the missing SNPs specific to or with higher frequencies in the AFR population?

We have re-run our analyses with a shared set of markers, and included these details in a supplemental table (Table S2.). The Results section now mentions that our broad conclusions are robust to whether the “optimal” proxies or “shared” proxies are used.


21. "Mean counts of risk alleles for each study site were used to fill any missing genotype data". What was the rate of missingness among the different populations?

Missing data was a rare occurrence. ~0.67% of all the PRS variants were missing for each sampled individual (regardless of population). We now include this detail in the Methods section of our paper.
Schumacher PRS: MADCaP missingness = 0.57%, UKBB missingness = 0.80%
Conti PRS: MADCaP missingness = 0.64%, UKBB missingness = 1.01%
PHS46+African PRS: MADCaP missingness = 0.47%, UKBB missingness = 0.51%


22. "Two-sided binomial tests were used to infer if SNPs contribute more to SNP heritability in Europe than in SSA (i.e., whether European or SSA allele frequencies are closer to 0.5)." MAF is not the only contributor to SNP heritability. This is really just a comparison of whether the allele frequencies are closer to 0.5, and should be stated as such.

Correct. We have re-worded the text to reflect this (plus added a citation).


23. The odds ratios should be calculated from logistic regression, not from the counts of cases and controls. The regression models should adjust for age and other covariates (including principal components for ancestry).

We have opted to retain our original way of calculating OR from counts of cases and controls (albeit with the suggested change of comparing the top decile to the middle 20% of PRS distributions). One reason for this is that available covariates differ between MADCaP and UKBB datasets. An additional complication is that using age as a covariate could impact tests of PRS effectiveness re: aggressive early-onset CaP. That said, future MADCaP Network studies are likely to delve deeper into age-stratification and fine-scale ancestry (including approaches that leverage chromosome painting).


Reviewer #2: The manuscript by Kim et al. is an important investigation how reliable are polygenic risk scores (PRS), which are still largely biased towards European-decedent data, to predict risk of prostate cancer and of aggressive forms of prostate cancer in males of African ancestry. They demonstrate how incorporation of ancestry-informed markers in the PRS do improve accuracy of inferences (not so much for the aggressive form), but, nonetheless, how far we still are of having identifying essential risk markers for the African ancestry. It is not surprising that PRS inferences within African work better for those of western African background, given that most information we have are for African-Americans, whom are mainly of western African ancestry, or from MADCaP excess of samples from western Africa. I agree that the publication of this manuscript is currently important, and raises awareness of how important it is to fund international collaboration with teams across Africa, as the MADCaP example. I have the following suggestions that can help improving the manuscript:

1- My main concern is about the evaluation of selection on the rs5919393, rs61088131, rs7888856, and rs11672691 markers. As the authors have demonstrated, the phenotypes under evaluation are under the effect of the interaction between many genes - if there is some selection effect, it is probably polygenic selection. So, it is highly improbable that those 4 markers were under the effect of a strong selective sweep. To apply just one algorithm that is only able to detect strong selective sweep is currently quite limiting. Check, for example, the calculation of "polygenic selection of complex traits" methods section of the manuscript by Lopez et al. 2019. Current Biology 29: 2926-2935.

We agree with Reviewer #2 that the likelihood that these loci are under strong selection is quite low. Along these lines, we have edited the text of the Results and Discussion sections to highlight neutral evolutionary processes as an explanation for large allele frequency differences at CaP-associated loci. Following Reviewer #2’s suggestion, we have conducted additional tests of polygenic selection. Specifically, we have run PolyGraph on sets of CaP-associated loci. Results of these analyses are shown in the newly renumbered Fig. 2c. Generally speaking, signatures of polygenic selection are negligible for each PRS analyzed here.


2- Another important aspect that the authors should be more careful in explaining and evaluating is the imputation of the genotypes of the PRSs. Imputation is not so reliable for African populations, given the huge bias in complete genome sequences between European and African individuals. What was the ratio of markers that needed imputation between European- and African-associated CaP markers? This is an important information for the Results section, and should not be left only for the Material and Methods section.

This is a good point, and it is particularly salient given differences in LD patterns across global populations. Unfortunately, using imputed proxies is unavoidable due to heterogeneous sets of markers on different genotyping platforms. See below for some statistics (now included as a tab in Table S1 of our revised manuscript). One thing to be keep in mind is that scenarios where proxies were unable to found tended to coincide with effect alleles being rare. Also recall that when proxies were used, their effect sizes were down-weighted. As per the suggestion of Reviewer #2, we mention the use of proxy variants in the Results section, and that proxies were used more often for the MADCaP dataset. We also mention that imputation issues can contribute to differences in PRS effectiveness in the Discussion section.
Schumacher PRS: MADCaP proxies = 15.1%, proxies = 7.5%
Conti PRS: MADCaP proxies = 38.1%, UKBB proxies = 6.7%
PHS46+African PRS: MADCaP proxies = 38.8%, UKBB missingness = 10.2%


3- Results section, page 9, after authors describe the "case/control ratios among individuals with high risk scores (population-specific PRS percentiles above 90%) to case/control ratios among individuals with low risk scores (population-specific PRS percentiles below 10%)", I got lost when they say "the Schumacher PRS yielded an OR of 9.83 for individuals from the UKBB and an OR of 1.36 for individuals from the MADCaP Network" that these values would mean (individual) ORs or those ratios. I think that another nomenclature that not simply OR should be used here, for clarification purposes. I immediately think about the OR at individual level - this is even worse if only looking at Table 2. The message it transmits is totally wrong - risk of CaP in Europeans would be huge compared to Africans.

We have edited the text for clarity, and have made it more explicit that these odds ratios are a measure of how effective a PRS is at able to be distinguish between cases and controls (as opposed to the relative risk of CaP for different ancestries). A sidenote: our initial analyses of PRS effectiveness focused on AUC statistics, but we opted to include ORs as well to be more in line with other related papers (e.g., Mavaddat et al. 2019 and Conti et al. 2021).


4- Results section, page 9, please give more information on the "ancestry-specific weights". What are these? I did not find any information even in Material and Methods. But definitely, it should be included in both sections. I had to go to Supplementary Table to check this - I understood that the weighing is by using different beta calculated in various ancestry groups of Conti dataset. Clearly state so, and provide information on sample sizes for each group.

Reviewer #2 is correct that the weightings use different betas for different ancestry groups of the Conti dataset. We have added two additional sentences along these lines to our Methods section, including a reference to Supplemental Table S4 in Conti et al. 2021.


5- In Material and Methods, Page 14/15, what do you really mean when you say "Mean counts of risk alleles for each study site were used to fill any missing genotype data"? If I am understanding correctly, when a genotype for a marker is missing for one individual, your estimate the mean frequency in the population for the risk allele and include that information instead of considering it a missing genotype? I do not agree with this. This is even worse for markers relevant for African ancestry-associated risk, as we do not have so much information about allele frequencies. The authors should at least state how much missing information did they allow for the individuals. If they did not include any restriction, maybe they have individuals for whom the PRS was based on 80%, 50%, 40% (???) inferred genotypes. How biasing was this additional factor in their analyses? Remember that this adds up to the fact that many genotypes had to be input.

The reason why we corrected for missing data was to avoid the scenarios where samples with greater amounts of missing data ended up having lower PRS values simply because there were fewer markers to sum. Fortunately, overall missingness rates were low: 0.67% on average (also see the table in response to query 21 from Reviewer #1). Individual samples were also excluded if they have over 5% missingness. On the whole this means that missing data at PRS markers had a negligible effect on our results. We hope that this alleviates any concerns of Reviewer #2. In any case, missingness statistics are now included in the Methods section of our revised manuscript.


6- In Figure 2, further information is needed for the "American" 1000Genomes group. Does it include "African ancestry in SW USA - ASW" and "African Caribbean in Barbados - ACB"? If yes, it does not make sense.

Good point. ASW and ACB individuals were included in the African group, as opposed to the American group. This detail is now mentioned in the figure legend.


Reviewer #3: This study applies polygenic risk scores to a case control data set from sub-Saharan Africa to see whether performance differs within the African dataset from performance within a comparably sized random sample of European data drawn from the UK biobank. The question of performance in diverse data set is an important one, especially for prostate cancer, where prior work has shown the potential clinical utility of predicted genetic risk to guide screening decisions. The dataset used here is particularly interesting because it is drawn directly from several areas of Africa. The authors are to be commended for collecting this African data set and for considering generalization performance from several angles.

1. It would be helpful to have any available additional detail about the characteristics of the individuals including in this study. For example, how much admixture is observed in these populations? South Africa, for example, is known to have multiple ethnicities. Can a brief introduction be given for the populations included from each site?

Done. We now include a new subsection of the Results that describes the population structure of the MADCaP Network dataset. An additional figure has also been added (showing 2D MDS plots and an ADMIXTURE plot). Note that MADCaP sample accrual focused on individuals of African ancestry (e.g., individuals of mixed ancestry from Cape Town were excluded if < 75% of their DNA did not have a recent African origin). We also list the most common languages spoken by MADCaP study participants in the revised version of our manuscript.


2. The finding that odds ratios in MADCaP are much smaller than in UK bio bank is important. Indeed, the difference in performance appears much larger between these two data sets than was reported previously for prostate cancer (PMID: 33792693, PMID: 33623038). The conclusion for the present study is written, "genetic prediction of prostate cancer risks perform poorly if the study sample does not match the ancestry of the original GWAS." It appears, however, that sometimes scores will perform relatively better or worse out of the original sample. This is both important to know and a suggestion that there is work to be done to understand what drives this variance in performance and how to predict it. The discussion and conclusion would be more informative with this additional context.

One thing to keep in mind is that 95% confidence intervals for odds ratios are pretty wide if sample sizes are small. Regarding the two papers mentioned above: Plym et al. (PMID:33792693) analyzed 67 African ancestry cases and 457 controls. Due to this small sample size 95% CI for their African ancestry OR90/50 ranged from 1.48 to 10.19. By contrast, Huynh-Li et al. (PMID: 33623038) analyzed 3,240 cases and 3,013 controls of African descent. This may explain why the Plym et al. paper found smilar OR for African samples and European samples while the Huynh-Li paper found much significantly smaller HR for African samples than European samples (compare Table 1 of Plym et al. Table 1 of Huynh-Li et al.). Due to the small African sample sizes of the Plym et al. paper, it makes sense to be cautious about claims that scores can perform better outside of the original sample. A recent study that examined 245 polygenic traits found that PRS effectiveness decay with genetic distance to the source GWAS, and we now mention this in the Discussion section (Privé et al. 2022 AJHG). That said, an additional possibility is that PRS that work particularly well for their source population might actually be overfit, and might have reduced portability. Put another way: are there trade-offs between effective prediction in the source population vs. the generalizability of PRS?


3. If available, more information should be given about how these cases were obtained. Is there commonly any early detection with PSA testing, or are these generally patient presenting with suspicious symptoms? The age range of the included individuals is fairly advanced, which could be noted in the text.

In general, African cases first present with symptoms, as opposed to being identified via PSA screening. This does indeed contribute to the age range and high Gleason Scores that are observed in our dataset. We now include a mention of this in the Methods section.


4. For completeness, please add confidence intervals for the disease severity analyses.

Done.


5. (Minor) Any thoughts as to why the continental differences in score distributions were smaller for the 46-variant score? Is it possible that variants with smaller effect sizes are more likely to be differentially distributed across populations?

Yeah, that is an intriguing hypothesis. One detail that is consistent with that idea is that rare alleles are more likely to be younger population-specific polymorphisms. Some of the observed patterns might simply be due to the PHS46+African PRS having smaller numbers of markers than the Schumacher and Conti PRS.


6. (Minor) The last paragraph on page 9 (lines 49 through 55) mentions that European versus multi ancestry weights performs better for AUC or odds ratio, respectively. This is of unclear significance. Confidence intervals should be reported in the text, at least, even if no further explanation can be given.

We agree with Reviewer #3. As seen in Table 1, the confidence intervals for the multi-ancestry and European weights overlap. In light of this, we have cleaned up this section of the text a little.
Also note that we have re-run our odds ratio analyses (as per the suggestion of Reviewer #1) by comparing the top 10% to the middle 20% of each PRS distribution.

**Second round of review**

**Reviewer 1**

The authors have done a nice job responding to my comments. I have one outstanding concern, based on my below request to adjust for covariates. I'm not sure I've seen a genetic association study not adjust for covariates (e.g., principal components). The explanation that the covariates differ or age may confound results does not seem sufficient. If anything, the adjusted results should be the primary findings, and the crude results could be listed as well. But only including the crude results would not seem acceptable.

23. The odds ratios should be calculated from logistic regression, not from the counts
of cases and controls. The regression models should adjust for age and other
covariates (including principal components for ancestry).
We have opted to retain our original way of calculating OR from counts of cases and
controls (albeit with the suggested change of comparing the top decile to the middle
20% of PRS distributions). One reason for this is that available covariates differ
between MADCaP and UKBB datasets. An additional complication is that using age as
a covariate could impact tests of PRS effectiveness re: aggressive early-onset CaP.
That said, future MADCaP Network studies are likely to delve deeper into agestratification and fine-scale ancestry (including approaches that leverage chromosome
painting).

**Reviewer 2**

The authors addressed successfully all my points. I agree with the publication as it is.

**Reviewer 3**

I am satisfied with the authors' thoughtful responses to reviewer comments.

**Authors’ response to reviewers**

We are pleased to submit a newly revised version of “Testing the generalizability of ancestry-specific polygenic risk scores to predict prostate cancer in sub-Saharan Africa” to Genome Biology. This manuscript introduces a novel dataset of 2,631 African prostate cancer cases and controls, and it tests of whether the inclusion of ancestry-matched information improves polygenetic predictions.

Once again we would link to thank the editors of Genome Biology and three anonymous reviewers for their helpful comments and suggestions. As requested, the revised version of our manuscript now includes odds ratio calculations that correct for age and population structure (the first 10 PCs). Importantly, the main conclusions of our manuscript remain the same: use of ancestry-matched data improves polygenic score performance. We have the updated odds ratios mentioned in the abstract, main text, and tables of our paper to reflect these new analyses. A slight re-ordering was made to the author list of this paper - to reflect Ujani Hazra’s extra effort in calculating covariate-adjusted odds ratios. Specific responses to reviewer comments are listed below.


Reviewer #1: “The authors have done a nice job responding to my comments. I have one outstanding concern, based on my below request to adjust for covariates. I'm not sure I've seen a genetic association study not adjust for covariates (e.g., principal components). The explanation that the covariates differ for age may confound results does not seem sufficient. If anything, the adjusted results should be the primary findings, and the crude results could be listed as well. But only including the crude results would not seem acceptable.”

We appreciate these positive words from Reviewer #1. The suggestion of re-running our polygenic risk score analyses after correcting for covariates is a good one. With this in mind, we recalculated odds ratios after correcting for age and the first 10 principal components using generalized linear models in R. Covariate-adjusted odds ratios were calculated for each combination of PRS and study population (see Table 2 and Table S2 for these values). For completeness, we also calculated covariate-adjusted odds ratios for aggressive vs. non-aggressive prostate cancer (found in the new supplemental Table S3). In general, adjusting for covariates had only a negligible effect on odds ratios for MADCaP data. However, adjusting for covariates yielded modest improvements across the board for UK Biobank data. That said, there were no qualitative changes in our conclusions. For example, the multi-ancestry and European weights for the Conti PRS still performed better for UK Biobank data than African, Asian, or Hispanic weights.


Reviewer #2: The authors addressed successfully all my points. I agree with the publication as it is.

We appreciate these positive words from Reviewer #2.


Reviewer #3: “I am satisfied with the authors' thoughtful responses to reviewer comments.”

We appreciate these positive words from Reviewer #3.

“The logistic regression strategy also permits incorporation of covariates, as requested by the reviewer. I also agree that this is generally recommended. Age is particularly important (and is the reason I prefer Cox survival methods for these analyses in my own work). The issue for this paper is going to be that there are probably starkly different prostate cancer detection patterns across the two datasets—e.g., the African dataset may have later age of diagnosis and correspondingly higher proportions of high grade / advanced stage at diagnosis. These are all murky questions with the available data, but the reviewer is correct that they are of interest. I do not think the authors’ response adequately dismisses these concerns. Early-onset disease is a minority of cases and less of a concern than the reviewer’s points. Lack of consistency in available covariates is a practical concern but hardly precludes using the covariates that are in common (and listing those that one would want to include but cannot)."

As per reviewer suggestions, we have re-run our odds ratio analyses after correcting for age and the first 10 principal components (see above for details). Once again, our primary conclusion remains the same: genetic predictions of prostate cancer perform poorly if the study sample does not match the ancestry of the original GWAS. Given the limitations of available datasets, further down-sampling MADCaP and UK Biobank data to yield identical age distributions would have greatly limited our statistical power to compute accurate odds ratios.

Thank you for your consideration.

Sincerely,
Joseph Lachance, on behalf of the authors
